# Anxiety Severity, Perceived Risk of COVID-19 and Individual Functioning in Emerging Adults Facing the Pandemic

**DOI:** 10.3389/fpsyg.2020.567505

**Published:** 2020-12-07

**Authors:** Alessandro Germani, Livia Buratta, Elisa Delvecchio, Giulia Gizzi, Claudia Mazzeschi

**Affiliations:** Department of Philosophy, Social Sciences and Education, University of Perugia, Perugia, Italy

**Keywords:** anxiety, emerging adulthood, instability, isolation, quarantine, risk perception, SARS-CoV-2

## Abstract

The COVID-19 pandemic is showing a strong impact on people in terms of uncertainty and instability it has caused in different areas of daily life. Uncertainty and instability are also emotions that characterize emerging adulthood (EA). They generate worries about the present and the future and are a source of anxiety that impacts negatively on personal and interpersonal functioning. Anxiety seems a central effect of the pandemic and recent studies have suggested that it is linked to COVID-19 risk perception. In the present study, a sample of 1045 Italian emerging adults was collected: (1) to assess anxiety severity and perceived risk related to COVID-19 and their association and (2) to compare general health and protective factors such as attitudes about security, relationships, self-esteem, and self-efficacy across anxiety severity and perceived risk categories. The findings of this study highlighted that anxiety severity categories were distributed homogeneously across the sample and that half of the participants referred to moderate-severe anxiety. A series of analysis of variances and *post hoc* comparisons showed that general health and all protective factors decreased according to anxiety severity. They were higher in participants with high perceived risk, with the exception of self-efficacy. Given the challenging features of the pandemic and EA, it is crucial to monitor anxiety severity in order to prevent last longing effects on mental and physical health, as well as keeping emerging adults informed about the risks related to the pandemic. Intervention and supportive programs based on improving self-esteem and self-efficacy, as well as confidence in relationships, should be offered to emerging adults over the long term, beyond the current outbreak.

## Introduction

The Coronavirus disease (COVID-19) (WHO) is a respiratory infection caused by a novel coronavirus named 2019-nCoV or SARS-CoV-2 or HCoV-19 (Jiang et al., 2020). In the beginning, it was identified in China where it caused an epidemic that started in December 2019 (Wu et al., 2020; Zhou et al., 2020), since which time its incidence has increased exponentially. Within months, it was declared a global pandemic by the World Health Organization. Italy was one of the first countries hit and one of the most affected by the pandemic in terms of morbidity and mortality (Dong et al., 2020; Johns Hopkins University and Medicine, 2020).^1^ The health system in Italy was, at one point, overwhelmed, with full intensive care departments. In response to the growing pandemic of COVID-19, on March 11, and March 21, 2020, the Italian Government imposed a national lockdown and all individuals were quarantined and forced to maintain strict social distancing from other people.

Uncertainty, insecurity, and instability in different areas of daily life including health, work and relationships were common feelings during the pandemic (Germani et al., 2020a). Moreover, they were exacerbated by scant and inconclusive information about 2019-nCoV and its deep social and economic impact (Ornell et al., 2020). Borrowing the title of a recent paper (Briem, 2020), we can say that during “COVID-19. The only certainty is the uncertainty.” Previous research emphasized the negative impact of uncertainty, insecurity, and instability on mental health and psychological well-being (Ornell et al., 2020; Torales et al., 2020). They underlined that uncertainty and the low predictability of COVID-19 personal, social, and financial consequences, are affecting people’s mental health, due to their difficulty to react successfully and satisfactorily to the situation, especially with regard to anxiety management (Jeong et al., 2016; Cao et al., 2020; Li et al., 2020a,b; Li S. et al., 2020; Wang et al., 2020). In a pandemic, fear and insecurity increase anxiety levels in healthy individuals and intensify the symptoms of those pre-existing psychiatric disorders (Shigemura et al., 2020). The number of people whose mental health is affected tends to be greater than the number of people affected by the infection (Reardon, 2015).

Studies among the Chinese general population have found that symptoms of anxiety have shown a higher increase due to COVID-19 (Li et al., 2020a,b; Wang et al., 2020). In one study, about a third of the interviewees reported moderate to severe anxiety, while they reported lower depressive and stress scores (Wang et al., 2020). In another study (Li S. et al., 2020), the most relevant change between before and after the declaration of the outbreak referred to anxiety levels, followed by depression and problems sleeping. Anxiety seems to be the central effect for populations throughout the world during the pandemic, which is raising anxiety levels in many countries (Fardin, 2020; Limcaoco et al., 2020).

However, anxiety is not always negative. It is important to highlight that it might have a positive effect on the preventative health behavior of individuals, which is strictly related to perceived risk (Brewer et al., 2007; Weinstein et al., 2007; Lin et al., 2020). Risk perception refers to people’s evaluation of the hazards that they are exposed to (Cori et al., 2020). An accurate perceived risk is crucial for managing public health risks during a pandemic because it is potentially a strong modifier of the evolution of the epidemic, since it could influence the number of new positive cases (Rogers, 1975; Ferguson, 2007; Cowling et al., 2010; Ibuka et al., 2010; van der Weerd et al., 2011; Cori et al., 2020; Dryhurst et al., 2020).

Anxiety and perceived risk, require special attention in emerging adults during the COVID-19 pandemic. Emerging adulthood (EA) was defined as the transition period of life from adolescence to adulthood (Arnett, 2000). Because emerging adults have more autonomy than adolescents and can change their lives in terms of work, residence, and relationships, EA is considered an age of identity exploration, a stage in life where there are many possibilities. However, EA is also characterized by instability and it is a very critical period (Arnett, 2000, 2004). Emerging adults are often fraught with precarity and worry about the future (Côté, 2014; Schwartz, 2016). If, on the one hand, psychological well-being can improve during this period (Galambos et al., 2006), many emerging adults are, on the other, more vulnerable to a worsening of the symptoms of anxiety, which can impact negatively on personal and interpersonal functioning (Kessler et al., 2005; Arnett, 2007; Schwartz, 2016). Moreover, EAs engage in more risky behaviors than adults (Nelson and Barry, 2005).

At the beginning of the pandemic, experts and social media emphasized that emerging adults were not so likely to catch COVID-19, which was considered similar to the flu and thought to be a disease of the elderly (Liao et al., 2020). In line with this, in many countries, Italy included, younger people paid less attention than others to COVID-19 (Barari et al., 2020; Van Bavel et al., 2020). The news and the media reported less preventative health behavior in EA, and some people ignored advice that people practice social distancing and stay at home. When the Italian government imposed the national lockdown, specifically in the period March 18–20, Italian emerging adults (aged 18–30) practices significantly less social distancing compared to adults and the elderly and left home for non-essential reasons more often than others. Italian emerging adults reported anxiety levels lower than in other stages of life, suggesting that they had no accurate risk perception (Barari et al., 2020). However, since this time, the findings indicate that the younger population has been increasingly affected by the virus, as well as other aspects related to the pandemic, and measures implemented by the government, and there are reports that younger participants experienced higher levels of anxiety than older participants (Casagrande et al., 2020; Rossi et al., 2020). This result is in line with findings from studies on the Chinese population, which confirmed that after 10–25 days of lockdown, younger participants (<35 years old) reported significantly higher levels of anxiety than older ones (Huang and Zhao, 2020). Anxiety severity and duration predict long-term outcomes and are linked to crucial aspects of psychological functioning in EA. Moreover, Germani et al. (2020a) found that Italian emerging adults reported higher levels of anxiety than the normative sample (Pedrabissi and Santinello, 1989). At the same time, Germani et al. (2020a) showed that Italian emerging adults were aware of the severity of the COVID-19 pandemic and they were worried and concerned about it. This study (Germani et al., 2020a) therefore suggested that after an early phase of the epidemic from which emerging adults seemed to be exempt (Liao et al., 2020), there is a growing number of positive cases and first deaths in youths, the awareness-raising implemented by all institutions and media, and the measures taken to fight the pandemic, have affected Italian emerging adults.

This developmental phase of life can seriously impact general health status, the perception of one’s value, a sense of personal competence, and security versus insecurity in relationships (Cozzarelli et al., 2003; Galambos et al., 2006; Arnett, 2016). Self-esteem (Rosenberg, 1965) is an important predictor of healthy development. Low self-esteem predicts poor mental and physical health, income, delinquency, and depression (Trzesniewski et al., 2006; Orth et al., 2009). A secure attitude in relationships is another crucial factor in EA, both in terms of changes in interpersonal relationships that occur in that period and of social cognition development (Lapsley and Woodbury, 2016; Tanner, 2016). In addition, it has a role in successful adaptation, mental health outcomes, and subjective well-being in EA (Bartholomew and Horowitz, 1991; Lopez and Brennan, 2000; Surcinelli et al., 2010; Mikulincer and Shaver, 2012; Marganska et al., 2013; Germani et al., 2020b).

Research has shown that anxiety is related to personal and interpersonal functioning such as general health (Baksheev et al., 2011), secure attitudes in relationships (Ditzen et al., 2008; Riggs and Han, 2009; Surcinelli et al., 2010; Marganska et al., 2013), self-esteem (Riggs and Han, 2009; Keane and Loades, 2017), and self-efficacy (Muris, 2002; Scholz et al., 2002). A secure attitude in a relationship, self-esteem, and self-efficacy, act as protective factors against anxiety related to the pandemic. They allow emerging adults to draw upon inner resources and to ask for help when they need it. Moreover, in general, emerging adults do not seem to have an accurate perception of risk, meaning they do not undertake preventative health behaviors in response the COVID-19 pandemic (Barari et al., 2020; Van Bavel et al., 2020). This could be related to personal and interpersonal functioning, since perceived risk and protective versus risk behaviors in the spread of infection are associated with self-esteem and self-efficacy (e.g., Golub et al., 2007) as well as to attitudes in relationships (e.g., Feeney et al., 2000).

In light of the above, it is relevant to evaluate anxiety severity and the perceived risk related to COVID-19 among Italian emerging adults. Moreover, it is of interest to test the association between anxiety severity and perceived risk with personal and interpersonal functioning. The present study aimed: (1) to assess anxiety severity and perceived risk related to COVID-19 and their association and (2) to compare general health and protective factors such as secure attitude in a relationship, self-esteem, self-efficacy across anxiety severity categories and perceived risk. It hypothesized that: (1) anxiety severity was higher than normative level and perceived risk related to COVID-19, indicating both a stressful reaction to the quarantine as well as a high risk perception; (2) that perceived risk partially increased according to anxiety severity, highlighting an association between them. However, considering the difference between these constructs, a small-moderate association was expected; and (3) on the one hand, emerging adults’ personal and interpersonal functioning becomes worse with anxiety severity, but on the other, it was better with an awareness of perceived risk about the pandemic, suggesting a positive link between high risk perception and personal and interpersonal functioning.

## Materials and Methods

### Participants and Procedure

The sample included 1045 Italian emerging adults (aged 18–29; Mn age = 24.18; SD = 3.60) from all over Italy. About 30% of the sample were male, around the same percentage were workers. About a third of participants reported a history of psychiatric/psychological disorders, and a small percentage (6.5%) disclosed a history of chronic physical diseases. Table 1 reports data on confirmed cases and health behaviors according to the main containment measures.

**TABLE 1:** Descriptive Analysis: Percentage of COVID-19 confirmed cases and health behavior and mean (±SD) scores and Cronbach’s alpha of the measures analyzed.

	***N***	**%**
**COVID-19 Confirmed cases**		
Participants	0	0
Participants’ relatives and/or friends	246	23.5
**COVID-19 Health behaviors**		
Stay at home	877	83.7
Social distancing	1036	99.2
Better personal hygiene	995	95.6

	**Mn ± Sd**	**α**

STAI-State	48.53 ± 12.64	0.94
COVID-19 PR	3.85 ± 0.69	0.65
RQ-SA	3.99 ± 1.85	
RSES	29.38 ± 6.34	0.89
GSE	28.38 ± 5.15	0.88
GHQ	16.24 ± 0.62	0.80

*STAI, State and Trait Anxiety Inventory; PR, Perceived Risk; RQ-SA, Relationship Questionnaire – Secure Attitude; RSES, Rosenberg’s Self-esteem; GSE, General Self-Efficacy; GHQ, General Health Questionnaire.*

Participants filled in an online survey form from March 17 to 26, 2020. Data was collected through convenience sampling. The inclusion criteria were: (1) agreed to participate after reading the study description; (2) that they were aged between 18 and 29 years; and (3), that they completed the entire online survey form.

We obtained approval from the Ethics Committee approval from the Department of Philosophy, Social Sciences, and Education, University of Perugia (Italy). Participation was voluntary, anonymous, and no incentive reward was given. All participants were given the optuion to withdraw at any moment.

### Measures

#### COVID-19 Perceived Risk (PR)

Participants indicated on a five-point scale (from 1 = “not at all” to 5 = “very much”) the risk perceptions to whom they were or could be exposed to, replying to the following questions: In general, how serious do you think COVID-19 is? How much are you worried about being infected with COVID-19? How worried are you about infecting your relatives?). PR was the average score of the three items.

#### State and Trait Anxiety Inventory-Y – State Scale (STAI-Y, Spielberger, 1989)

We used 20 items assessing how prone each participant was to anxiety in a specific moment. All the items were rated on a 4-point Likert scale from 1 (not at all) to 4 (very much so). Total scores ranged from 20 to 80, with higher scores, indicating a higher level of anxiety. Previous studies showed the S*tate Scale* is a reliable measure with good convergent and discriminant validity (Spielberger, 1989). The Italian version of the STAI-Y State Scale was used (Pedrabissi and Santinello, 1989).

#### Relationship Questionnaire – Secure Attitude (RQ, Bartholomew and Horowitz, 1991)

We used a questionnaire asking participants to describe their secure attitude in a relationship, which had the following options: “It is easy for me to become emotionally close to others. I am comfortable depending on others and having others depend on me. I don’t worry about being alone or having others not accept me.” Participants were asked to rate their attitude from 1 (does not describe me at all) to 7 (describes me exactly). The RQ showed adequate psychometric properties (Ravitz et al., 2010). The Italian version of the RQ (Fossati et al., 2007) was administered.

#### Rosenberg’s Self-Esteem Scale

Rosenberg’s Self-Esteem Scale (RSES, Rosenberg, 1965) is a 10-item self-report measure for evaluating the self-worth with a 4-point Likert scale from 1 (Strongly Disagree) to 4 (Strongly agree). A higher score indicates higher self-esteem. RSES showed adequate internal consistency and its validity has been demonstrated in different cultures and languages (Schmitt and Allik, 2005). The Italian version of the RSES (Prezza et al., 1997) was used.

#### General Self-Efficacy Scale (GSE, Schwarzer and Jerusalem, 1995)

This scale evaluates an individual’s self-efficacy. It is composed of 10 items, scored on a 4-point scale from 1 (not al all true) to 4 (exactly true). The higher the score the higher the self-efficacy. GSE psychometric characteristics have been extensively studied across several countries by Scholz et al. (2002). The Italian version (Sibilia et al., 1995) was used.

#### General Health Questionnaire -12 (GHQ-12, Goldberg and Blackwell, 1970)

This questionnaire assesses general and psychological distress through 12 items rated on a 4-point Likert scale from 0 (less than usual) to 3 (much more than usual). Lower scores indicate lower distress and better general health. A previous study showed GHQ-12 as a reliable and valid measure (Werneke et al., 2000). The Italian version of the GHQ-12 (Piccinelli et al., 1993) was used.

### Data Analysis

Descriptive statistics in terms of mean, standard deviation, and percentage were run for describing, anxiety, and PR related to COVID-19. Four categories of anxiety severity were created according to STAI-Y clinical cutoff (Knight et al., 1983): (1) scores <40 indicate low anxiety symptoms, (2) scores from 40 to 50 indicate mild anxiety symptoms, (3) scores from 51 to 60 indicate moderate anxiety symptoms and finally (4) scores >60 indicate severe anxiety symptoms. Furthermore, two categories of PR were created by using the median (50th percentile).

Internal consistency was calculated based on Cronbach’s alpha. According to Vaske (2008), alpha values between 0.65 and 0.80 are considered “adequate” for scales adopted for research on human dimensions. Pearson correlation was performed to analyze the association between anxiety severity and PR. A series of Univariate Analysis of Variance (ANOVA) were run to compare secure attitudes in a relationship, self-esteem, self-efficacy, and general health across anxiety severity and PR categories, controlling for the potential interaction between them. The effect size was measured using partial eta-squared, in which small, medium, and large effects were.0099, 0.0588, and.1379, respectively (Cohen, 1988, p. 283). *Post hoc* comparisons with Bonferroni correction were conducted for anxiety severity categories. All analyses were performed using SPSS, release 25 (IBM Corp, 2017).

## Results

Descriptive statistics and the internal consistency of each of the variables were reported in Table 1.

Results showed that 17.6% (*n* = 184) of emerging adults referred to severe levels of state anxiety, 25.8% (*n* = 270) moderate levels, 28.4% (*n* = 297) mild levels, and then 28.1% (*n* = 294) reported low levels of anxiety related to COVID-19. Referring to PR, 536 participants (51.3%) were rated as Low PR, while 509 (48.7%) participants were rated as High PR. The Pearson correlation highlighted a positive significant relationship between state anxiety and PR (*r* = 0.255; *p* < 0.001).

The first ANOVA showed a significant main effect of anxiety severity [*F_(__3_,_1037__)_* = 16.72; *p* < 0.001; 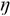
*_*p*_^2^* = 0.046] and PR (*F* = 6.47; *p* = 0.011; 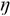
*_*p*_^2^* = 0.011) categories on secure attitude in relationships, while the interaction between them was not significant [*F_(__3_,_1037__)_* = 1.08; *p* = 0.358; 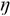
*_*p*_^2^* = 0.003]. *Post hoc* comparisons related to anxiety categories are shown in Table 2 and they indicate that a secure attitude in a relationship was the highest in low anxiety, followed by mild-moderate, and severe anxiety. As shown in Table 3, a secure attitude in relationships was higher in the High PR group than the Low PR group.

**TABLE 2:** Univariate Analysis of Variance (ANOVA) for severity anxiety categories with means and standard deviation scores of psychological features and general health status.

	**Low anxiety (1)**			**Mild anxiety (2)**			**Moderate anxiety (3)**			**Severe anxiety (4)**			***F*_(3,1037)_**	** 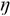 *_*p*_^2^***	***POST HOC***
	***n***	***M***	**SD**	***n***	***M***	**SD**	***n***	***M***	**SD**	***n***	***M***	**SD**			
**Psychological Features**															
RQ-SA	294	4.48	1.81	297	4.03	1.83	270	3.86	1.73	184	3.34	1.91	16.72**	0.046	1 > 2 = 3 > 4
RSE	294	33.39	0.32	297	30.00	0.32	270	27.89	0.33	184	24.20	0.41	116.41**	0.252	1 > 2 > 3 > 4
GSE	294	31.33	0.27	297	28.47	0.27	270	27.17	0.29	184	25.30	0.35	70.91**	0.170	1 > 2 > 3 > 4
**General Health Status**															
GHQ	294	13.26	3.86	297	15.62	3.49	270	17.45	3.53	184	20.22	3.82	147.15**	0.299	1 < 2 < 3 < 4

*RQ-SA, Relationship Questionnaire – Secure Attitude; RSES, Rosenberg’s Self-esteem; GSE = General Self-Efficacy; GHQ, General Health Questionnaire. *p < 0.01; **p < 0.001 indicates significant differences among severity anxiety categories. 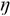
*_*p*_^2^* = partial eta-squares in which 0.0099 = small effect size; 0.0588 = medium effect size; 0.1379 = large effect size.*

**TABLE 3:** Univariate Analysis of Variance (ANOVA) for perceived risk about COVID-19 with means and standard deviation scores of psychological features and general health status.

	**Low PR**			**High PR**			***F*_(1,1037)_**	** 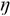 *_*p*_^2^***
	***n***	***M***	***SD***	***n***	***M***	***SD***		
**Psychological Features**								
RQ-SA	536	3.93	1.83	509	4.01	1.87	6.47*	0.015
RSE	536	29.36	6.31	509	29.41	6.37	14.01***	0.013
GSE	536	28.58	5.13	509	28.17	5.17	2.26	0.002
**General Health Status**								
GHQ	536	16.08	4.37	509	16.41	4.37	6.02*	0.006

*PR, Perceived Risk; RQ-SA, Relationship Questionnaire – Secure Attitude; RSES, Rosenberg’s Self-esteem; GSE, General Self-Efficacy; GHQ, General Health Questionnaire. *p < 0.05; **p < 0.01; *** *p* < 0.001 indicate significant differences among PR categories. 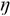
*_*p*_^2^* = partial eta-squares in which 0.0099 = small effect size; 0.0588 = medium effect size; 0.1379 = large effect size.*

The second ANOVA showed a significant main effect of anxiety severity [*F_(__3_,_1037__)_* = 116.41; *p* < 0.001; 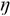
*_*p*_^2^* = 0.252] and PR [*F_(__1_,_1037__)_* = 14.01; *p* < 0.001; 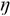
*_*p*_^2^* = 0.013] categories on self-esteem, while the interaction between them was not significant [*F_(__3_,_1037__)_* = 0.73; *p* = 0.534; 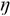
*_*p*_^2^* = 0.002]. *Post hoc* comparisons related to anxiety categories are shown in Table 2 and they indicated that self-esteem was highest in low anxiety, followed by mild, moderate, and severe anxiety. As shown in Table 3, self-esteem was higher in the High PR group than the Low PR group.

The third ANOVA showed a significant main effect of anxiety severity [*F_(__3_,_1037__)_* = 70.91; *p* < 0.001; 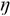
*_*p*_^2^* = 0.170] on self-efficacy, but not for PR [*F_(__1_,_1037__)_* = 2.26; *p* = 0.133; 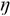
*_*p*_^2^* = 0.002]. The interaction between anxiety severity and PR was considered negligible due to its effect size [*F_(__3_,_1037__)_* = 2.88; *p* = 0.035; 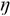
*_*p*_^2^* = 0.008]. *Post hoc* comparisons related to anxiety categories are shown in Table 2 and they indicated that self-efficacy was the highest in low anxiety, followed by mild, moderate, and severe anxiety.

The last ANOVA showed a significant main effect of anxiety severity categories on general health status [*F_(__3_,_1037__)_* = 147.15; *p* < 0.001; 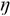
*_*p*_^2^* = 0.299]. The effect of PR was negligible [*F_(__1_,_1037__)_* = 6.02; *p* = 0.014; 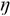
*_*p*_^2^* = 0.006] and the interaction between anxiety severity and PR was not significant [*F_(__3_,_1037__)_* = 1.33; *p* = 0.261; 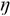
*_*p*_^2^* = 0.004]. *Post hoc* comparisons related to anxiety categories are shown in Table 2. They indicated that general health status was lower (less distress) in low anxiety, followed by mild, moderate, and severe anxiety.

## Discussion

Uncertainty for the future and anxiety are core feelings for both this pandemic (Jeong et al., 2016; Cao et al., 2020; Fardin, 2020; Li et al., 2020a,b; Li S. et al., 2020; Limcaoco et al., 2020; Ornell et al., 2020; Shigemura et al., 2020; Torales et al., 2020; Wang et al., 2020) and the developmental phase of the life that EAs experience (Arnett, 2000, 2004, 2007; Kessler et al., 2005; Côté, 2014; Schwartz, 2016; Germani et al., 2020a). Another crucial feature of EA is the low perception of risk, which leads some emerging adults to engage in more risky behaviors than adults (Nelson and Barry, 2005). Thus, they could represent one of the main categories of people who are vulnerable to the effects of the pandemic (Casagrande et al., 2020; Germani et al., 2020a; Huang and Zhao, 2020; Rossi et al., 2020). For this reason, the current paper explored emerging adults’ personal and interpersonal functioning taking into account anxiety severity and PR.

Italian emerging adults have reported high perceived risk about the pandemic. They rated it as severe, and they were aware of the elevated risk of being infected and infecting others, although none were directly or indirectly affected by COVID-19 (i.e., through relatives and/or friends). Moreover, their accurate perceived risk helped them in respecting the key containment measures imposed by the government, namely staying at home, maintaining social distancing, and washing hands (Brewer et al., 2007; Weinstein et al., 2007). On the other hand, it is reasonable to assume that those who underestimated the risk and severity of COVID-19, had scant knowledge and awareness of the pandemic or a denial of the risk, probably unconsciously (Cava et al., 2005; Larsman et al., 2012).

About half of emerging adults referred to moderate-severe levels of anxiety, suggesting very high distress. In response to the pandemic, they experienced a strong feeling of insecurity, of powerlessness in the face of perceived damage that can lead either to concern or to flight and avoidance. This finding is in line with previous studies that reported very high anxiety levels among emerging adults during the pandemic (Casagrande et al., 2020; Cellini et al., 2020; Huang and Zhao, 2020; Lin et al., 2020; Rossi et al., 2020). Specifically, we found an average score very close to the one found by Lin et al. (2020) among Chinese, who assessed state anxiety with STAI-Y. However, Cellini et al. (2020), who administered the short version of the Depression Anxiety Stress Scale which distinguishes normal, mild, moderate, severe, and extremely severe anxiety symptoms during the last week, and found that 32.6% of the sample reported moderate to extremely severe scores. Moreover, Rossi et al. (2020), used the Generalized Anxiety Disorder scale in Italian adults (Mage = 38 years old) and found that 20.8% reported severe anxiety symptoms. Although the number of participants who reported moderate-severe anxiety in the present study is higher than the ones from the aforementioned studies, due to the different questionnaire administered to them and/or the different age range of the participants, it is not possible to compare these results. Finally, anxiety was partly related to risk perception, in line with the literature (Lin et al., 2020), suggesting that it may have a positive effect on preventative health behavior (Brewer et al., 2007; Weinstein et al., 2007).

Personal and interpersonal functioning such as a secure attitude in relationships, self-esteem, and self-efficacy, as well as general health status, were linked to anxiety severity. The higher the anxiety severity, the lower the general health status and protective factors. These strong feelings and responses to the instability and uncertainty caused by the pandemic, seem to be strongly related to a negative model of self and others. In other words, low self-esteem and confidence in relationships were reported by participants with severe-moderate anxiety. The latter and low self-esteem are the main characteristics of the ruminative identity style in EA as well as intrusive thoughts, which expose to depressive symptoms (Crocetti et al., 2011). Attachment is considered a crucial factor for the challenges of EA, changes in the world of interpersonal relationships that take on new significance during EA, social cognition development, and subjective well-being (Arnett, 2016). Self-esteem is an important predictor of healthy development. Instead, emerging adults with positive self-esteem and trust in relationships can face the daily challenges by using their internal sources, as well as by asking for help when they need it (Murray et al., 2000; Germani et al., 2020b). Self-esteem increases from childhood to adolescence and reaches its peak in EA (Bleidorn et al., 2015). Thus, it is of crucial relevance to take this into account as a protective factor during the health emergency. This sense of self-confidence and confidence in others could help them in controlling their anxiety and enable awareness about the severity of the pandemic. It is noteworthy that we found larger effect sizes in the comparison between anxiety categories in self-esteem and general health status than in the other psychological features assessed.

Shifting to perceived risk, positive self-esteem, and a secure attitude in relationships were reported by emerging adults who showed a high PR, namely a more accurate understanding of the situation. In other words, this finding indicates that positive self-esteem and trust in relationships might have allowed emerging adults to fully perceive and feel the severity of the COVID-19 pandemic and preoccupation for risk infection and that a good and stable general perception of one’s-own value, as well as confidence in self and others, are key factors of emotional resilience in this age group.

The limitations of this study are connected to the generalizability of the findings due to the convenience sampling method, the direction of causality between the selected variables because this study is cross-sectional, and the common variance between variables due to the measurement method (i.e., self-report questionnaires). Moreover, the fact that the entire online survey had to be completed before participants were enrolled in the study, could potentially represent a further threat to generalizability.

To conclude, the present study indicated that during the first weeks of the quarantine, Italian emerging adults evaluated the pandemic as severe with a very high risk of infection, showing a realistic perception of the situation. Anxiety was a common feeling during those weeks and its severity was positively associated with perceived risk. Both self-esteem and a secure attitude in relationships seemed to protect EAs emotionally in this context.

This study recommends monitoring emerging adults and their psychological functioning throughout all the stages of the health emergency. COVID-19 has crucially affected emerging adults’ work, study, social lives, and caring responsibilities in a time of personal and interpersonal change. The various restrictions have exacerbated both economic and social inequalities. However, the current study underlined that a greater contributory factor to anxiety about this situation is connected to people’s ability to understand the severity of the virus. It suggests that it is important to track anxiety severity in emerging adults during the next steps of the COVID-19 pandemic, considering that high and prolonged anxiety exposes them to mental and physical disorders. This study suggests that continuing to keep emerging adults informed about the risks related to the pandemic will promote preventative health behaviors. At the same time, public health and health policy should track anxiety severity and help emerging adults in understanding and accepting the common psychological reactions to COVID-19 without avoiding and/or denying them and the related risks. Finally, mental health interventions such as psychological therapy sessions (i.e., psychodynamically and/or behavioral cognitive-oriented) and supportive programs based on maintaining and improving self-esteem and self-efficacy, as well as confidence in relationships, which aim to help them cope with anxiety related to the pandemic, should be offered to emerging adults over the long term, far beyond the current outbreak.

## Data Availability Statement

The raw data supporting the conclusions of this article will be made available by the authors, without undue reservation.

## Ethics Statement

The studies involving human participants were reviewed and approved by Ethics Committee of the Department of Philosophy, Social Sciences and Education of the University of Perugia (Italy). The patients/participants provided their written informed consent to participate in this study.

## Author Contributions

LB and GG organized the database and performed the statistical analysis. AG and ED wrote the first draft of the manuscript. LB and CM wrote the sections of the manuscript. All authors contributed to the conception and design of the study, manuscript revision, and read and approved the final version.

## Conflict of Interest

The authors declare that the research was conducted in the absence of any commercial or financial relationships that could be construed as a potential conflict of interest.

## Footnotes

1 https://www.arcgis.com/apps/opsdashboard/index.html#/bda7594740fd40299423467b48e9ecf6
